# What Is the Sense of Agency and Why Does it Matter?

**DOI:** 10.3389/fpsyg.2016.01272

**Published:** 2016-08-29

**Authors:** James W. Moore

**Affiliations:** Department of Psychology, Goldsmiths, University of LondonLondon, UK

**Keywords:** consciousness, free will, responsibility, human-computer-interaction, legal, aging, schizophrenia, OCD

## Abstract

Sense of agency refers to the feeling of control over actions and their consequences. In this article I summarize what we currently know about sense of agency; looking at how it is measured and what theories there are to explain it. I then explore some of the potential applications of this research, something that the sense of agency research field has been slow to identify and implement. This is a pressing concern given the increasing importance of ‘research impact.’

## Introduction

This article aims to serve two purposes. First, I wish to provide a general overview of research on sense of agency. This is by no means exhaustive, and is instead intended to give the reader a broad introduction to the topic. Second I wish to explore some areas in which this research may have some kind of impact. Impact is becoming an increasingly important issue for research scientists. According to the UK’s 2014 Research Excellence Framework, impact can be defined as research having an “an effect on, change or benefit to the economy, society, culture, public policy or services, health, the environment or quality of life, beyond academia.” Although many scientists are dubious of this increased focus on impact, it is almost certainly here to stay, at least for the foreseeable future. We all, therefore, would be well-served by being mindful of it in our own research. Some research topics lend themselves to impact more than others. Sense of agency falls more into the latter group; those working on this topic may struggle to articulate the relevance and potential impact of what they do. This article is, in part, an attempt to address this issue.

## What Is the Sense of Agency?

### Background

When we make voluntary actions we tend not to feel as though they simply happen to us, instead we feel as though we are in charge. The sense of agency refers to this feeling of being in the driving seat when it comes to our actions.

Synofzik et al. (2008) draw an important distinction between the *Feeling* of agency (FOA) and the *Judgment* of agency (JOA). FOA is a lower level non-conceptual feeling of being an agent; it is the background buzz of control we feel for our voluntary actions when not explicitly thinking about them. JOA, on the other hand is a higher-level conceptual judgment of agency, and arises in situations where we make explicit attributions of agency to the self or other. The FOA is linked to low-level sensorimotor processes, whilst the JOA to higher-level cognitive processes such as background beliefs and contextual knowledge relating to the action. These two levels of agency processing, although related, can be dissociated from one another. For example, an unexpected action outcome, which would signal non-agency at the FOA level, can nevertheless be attributed to the self at the JOA level if beliefs and contextual factors imply self-causation (for example, when an unexpected action outcome happens when acting alone in a room). This distinction between FOA and JOA also highlights an important distinction between agency and causality. Whilst the low-level FOA automatically registers agency or non-agency by tracking sensorimotor contingencies, the higher-level JOA deploys more general-purpose causal attribution processes that are not specifically linked to the sensorimotor system (see Gallagher, 2000, for a detailed discussion of this).

As with other aspects of conscious experience, the sense of agency is not an infallible reproduction of objective reality. As a consequence of this our experiences of agency can go awry. This is quite common in gambling, where players often feel an exaggerated sense of agency. An example of just such an illusion of control was noted by Henslin in the 1960s (Henslin, 1967). Henslin was a sociologist and spent a number of weeks observing cab drivers in St. Louis in the USA. A popular past-time among the cabbies was craps, a dice rolling gambling game. Henslin joined in with these games and made an intriguing observation. When these cab drivers came to roll the dice they altered their behavior depending on the number they needed, throwing harder for higher numbers and more gently for lower numbers. What is striking about this kind of behavior is that the outcome of dice rolling is objectively uncontrollable. Nevertheless these cabbies clearly felt otherwise. This is an example of where the sense of agency can be quite divorced from objective reality.

You might think that you are immune to such cognitive foibles, but you would almost certainly be mistaken. I would bet that most of us have fallen foul, at some point, of so-called ‘placebo buttons.’ These are buttons that we encounter every day that we think do things, but actually do nothing (McRaney, 2013). Buttons at pedestrian crossings are a common example of placebo-buttons. Most of these buttons are ineffective and instead the changing of the traffic lights are linked to timers. This was shown by a recent survey of pedestrian crossings in New York (McRaney, 2013). Intriguingly, most of us fail to notice the causal inefficacy of our button presses. Other examples of placebo buttons include ‘close door’ buttons in lifts and even thermostats in offices (many of which, apparently, do not work).

There are two reasons for flagging up the occasional lapses in our sense of agency. The first is to show that the accuracy of this experience is not a given. Instead, the brain appears to actively construct the sense of agency, and because of this, our experiences of agency can be quite divorced from the facts of agency. The second reason is that these lapses reveal something quite remarkable about our sense of agency: its impressive flexibility. Beyond the examples I have given here, we see over and over again that people come to experience control over outcomes in many weird and wonderful situations. The voodoo doll is another example; people taking part in this practice genuinely believe that sticking a pin in an effigy of someone causes actual physical harm in that person. At first blush this inference seems irrational. However, examples like the voodoo doll actually hint at the adaptability and flexibility of the agency processing system. It is worth reminding ourselves that causal mechanisms are quite opaque in a lot of modern technology (consider the simple act of tapping on a keyboard and seeing a letter appear on the screen in front of you – there are a lot of steps in this causal chain that are hidden from you). Despite this causal opacity, we feel in control of these interactions. So the flexibility that might make us vulnerable to agency errors in things like placebo buttons and voodoo dolls, can also allow our experience of agency to extend into new domains and track the rapidly changing agentic structure of our environment. Rather than our agency processing system breaking down with the development of tools, which have changed and extended our agentic capabilities, it has been flexible and adaptable, allowing us to accommodate these changes.

### Measures

The number of scientific investigations of sense of agency has increased considerably over the past 20 years or so. This increase is despite the fact that experiments on sense of agency face certain methodological problems. A major one is that the sense of agency is phenomenologically thin (Haggard, 2005). That is, when we make actions we are typically only minimally aware of our agentic experiences. This is quite unlike conscious experience in other modalities, especially vision, where our experiences are typically phenomenologically strong and stable. What this means is that sense of agency can be difficult to measure. As a result of this, experimenters have had to be quite inventive in order to develop paradigms that capture this rather elusive experience.

You can generally group these paradigms into implicit or explicit measures. Implicit measures assess a correlate of voluntary action and infer something about the agentic experience on the basis of this. In these paradigms no one is ever asked, directly, about their agentic experience. Probably the most widely used implicit measure of sense of agency is intentional binding (for a review see Moore and Obhi, 2012). This was developed by Haggard et al. (2002) and is based on time perception. Haggard et al. (2002) found that when we make a voluntary action, the perceived times of the action and its effect are shifted toward each other. This change in time perception is taken to be an implicit marker of sense of agency. Other implicit measures of sense of agency include sensory attenuation paradigms. It has been shown that the perceived intensity of the sensory consequences of voluntary action is lower than for passive movements (Blakemore et al., 1998, 1999). This can explain why we are unable to tickle ourselves (Blakemore et al., 1998). In these sensory attenuation paradigms, researchers use changes in perceived intensity of sensory feedback to infer something about the participant’s sense of agency.

Explicit measures, on the other hand, directly ask the participant to report something about their agentic experience. These measures are more intuitive but they can be vulnerable to problems like demand effects. A number of these paradigms require participants to make action recognition judgments. Typically the participant makes an action, but does not directly see that action. Instead they are shown some kind of feedback on a screen. This feedback may depict the participant’s action or it might depict the action of someone or something else (perhaps an experimenter or a computer), and the participant is asked whose movement it is. Importantly, the experimenter ensures that there is some uncertainty over the agent of the action being displayed. An example of this kind of task was used by Farrer et al. (2008). They had participants perform regular finger tapping movements while wearing a glove. They could not directly see these movements, and instead they were shown video feedback of the movement on the screen. A delay was inserted between the movement and the feedback presented to the participant. The participants were not aware that the movement was always their own, and instead were led to believe that the movement was either their own or an experimenter performing the same movement, and that this could switch at any time. The participant simply had to indicate when they thought they were seeing their own movement and when they thought they were seeing the experimenter’s movement. Farrer et al. (2008) found that participants experienced a bi-stable impression of agency in this situation, with judgments of agency spontaneously flipping between self and experimenter.

Other explicit measures also use visual feedback about movements, but will not create this kind of self/other confusion. Instead, the participant is required to make a judgment about the feedback itself. An example of this kind of action monitoring task can be seen in an experiment carried out by Synofzik et al. (2010). In this experiment participants made pointing movements under a screen, meaning that they could not directly see the movement. On the screen participants were shown a visual marker (white disk) that tracked the pointing movement. This marker was rotated by varying degrees relative to the actual movement. The participants had to indicate the direction in which the visual feedback was rotated relative to the actual movement. This gave the experimenters a measure of action awareness and, more specifically, sensitivity to distortions in action-relevant feedback.

A final kind of explicit measure requires participants to report on their feeling of agency for certain action outcomes that their movements might have caused. A simple example of this would involve a key press that causes an outcome after a variable delay. Participants would then judge how much they felt their action caused the outcome. A common finding is that such causal judgments are stronger for shorter delays (e.g., Shanks et al., 1989; Chambon et al., 2015). Interestingly, this kind of explicit measure taps into a slightly different aspect of the agentic experience compared with the other two kinds of explicit measure described in this section. Action recognition/monitoring tasks focus more on the action element, whereas, causal judgment tasks focus on the outcome component. Although, both of these are central to the agentic experience, this difference is often overlooked and not very well-understood.

### Theories

The two most influential theories of sense of agency have been the ‘Comparator Model’ developed by Frith et al. (2000) and Frith (2005), the ‘Theory of apparent mental causation’ developed by Wegner and Wheatley (1999) and Wegner (2002). The comparator model takes as its starting point the motor control system. We now know a great deal about the computational processes underpinning the control of voluntary movement (see Wolpert and Miall, 1996, for a review). According to the comparator model, some of these processes also inform the sense of agency. On this view, our actions start with intentions or goals, which enables a representation to be formed of the desired state of the motor system. Controllers within the motor control system then use this information about the desired states to generate a motor command. This motor command produces a movement, which changes the state of the motor system, and generates sensory feedback. On the basis of this information the new state of the system can be estimated. This estimate is compared with the desired state at a comparator. If there is a mismatch then an updated motor command is issued. This process can continue until the desired state is achieved (indicated by the absence of a mismatch at the comparator).

The issue with a motor system operating only in this way is that it is slow to respond to error. Because of this, the organism is vulnerable. The solution, it would appear, is to have an additional predictive component within the motor system, and it is this that is thought to be particularly relevant to sense of agency. This predictive component uses a copy of the motor command that is issued (a so-called ‘efference copy’) to predict the future state of the system. This includes predictions about changes to the motor system as well as the sensory consequences resulting from those changes. On the basis of these predictions, a representation of the predicted state of the system can be formed, and this representation can be compared both with the desired state of the system and with the actual state of the system. The former comparison is important for motor control, as it allows the organism to rapidly adjust motor commands in advance of incorrect actions being performed. The latter comparison is thought to be important for sense of agency. According to the comparator model, the output of the comparison between predicted and actual states determines whether or not we feel a sense of agency. If there is a match, then we feel a sense of agency; if there is mismatch then we do not.

A number of studies support this idea that sense of agency is closely tied to sensorimotor processes. For example, as predicted by the comparator model, it has been shown that the perceived intensity of self-produced tactile sensations is attenuated relative to externally produced ones (Blakemore et al., 1998, 1999). It has also been shown that sensorimotor prediction contributes to intentional binding (Moore and Haggard, 2008). Finally, mismatches between expected and actual sensory feedback influence action recognition judgments (e.g., Daprati et al., 1997; Franck et al., 2001).

The ‘Theory of apparent mental causation’ approaches sense of agency from a quite different angle. Whereas the comparator model places a heavy emphasis on the contribution of the motor system to sense of agency, the theory of apparent mental causation explicitly *downplays* this contribution. Indeed, according to this theory, it is because we do *not* have conscious access to the motor control system that our sense of agency can, at times, be so misleading, as seen in phenomena like voodoo dolls and placebo buttons. According the theory of apparent mental causation when we make a voluntary action there is an *unconscious* causal pathway that is responsible for the action. This pathway corresponds to the workings of the motor control system. There is also an unconscious causal pathway that is responsible for the associated thoughts about actions (i.e., intentions). In addition to these unconscious causal pathways, there are certain events that we are conscious of, namely the intention to act and the act itself. According to Wegner it is the relationship between the thought and the action that determines the sense of agency (or in Wegner’s term, the ‘experience of conscious will’). If our intention to act happens before we act, is consistent with the action, and is the only plausible cause of the action, then we feel as though we have caused the action. A fundamentally important feature of Wegner’s theory is the additional claim that this feeling is illusory – the inference that our intentions have caused our actions is erroneous (the unconscious pathways are the real causes of our actions.

A number of studies support Wegner’s theory of apparent mental causation. For example, it has been shown that priming thoughts about an upcoming action fosters an illusory sense of agency for that action (Wegner and Wheatley, 1999; Wegner et al., 2004). It has also been shown that manipulating high-level contextual information about an action (in the form of causal beliefs) alters sense of agency, as measured by intentional binding (Desantis et al., 2011).

These two theories, the comparator model and the theory of apparent mental causation, offer competing accounts of sense of agency. They differ in terms of the sources of information thought to be most important for producing sense of agency. For the comparator model, sensorimotor processes are key. For the theory of apparent mental causation, the emphasis, at least in the experimental work carried out to test it, has been on information that is external to the motor system, such as environmental and social cues (for a more extensive discussion, see Wegner and Sparrow, 2004). The traditional assumption has been, therefore, that these two views are mutually exclusive. However, this assumption has challenged by a number of studies. For example, using the intentional binding measure Moore and Haggard (2008) showed that both internal sensorimotor prediction and external action outcomes contributed to the sense of agency. It was found that binding of the action to the tone outcome was present when the probability of that outcome was high, even when it did not occur. This suggests that if sensorimotor prediction is sufficiently strong binding will occur. On the other hand, it was found that when sensorimotor prediction was weak, binding would occur but only when the key press actually caused the tone outcome. This would suggest that the presence of an external tone outcome retrospectively triggered the binding effect.

Findings such as these led us to develop an alternative ‘cue integration’ theory of sense of agency (Moore et al., 2009; Moore and Fletcher, 2012). This helped us move beyond the debate over whether sense of agency was based on sensorimotor information (comparator model) *or* information external to the motor system (theory of apparent mental causation). Instead, according to the cue integration theory both views have merit, and in fact the sense of agency is based on *various* different sources of information (or agency cues). We have also suggested that the relative influence of the different sources of information may be linked to their reliability, with the more reliable source of information dominating the agentic experience. We can see evidence of this in Moore and Haggard’s (2008) study, described above, where the influence of external action outcomes on intentional binding increased when the reliability of sensorimotor prediction decreased. We can also see evidence of this in patients with schizophrenia. Using an agency attribution paradigm, Synofzik et al. (2010) showed that agency judgments in people with schizophrenia relied more strongly on visual feedback about an action rather than on internal sensorimotor cues. This reliance on external visual feedback is consistent with the cue integration theory, as it has been shown that sensorimotor prediction is unreliable in people with schizophrenia (a similar finding was also obtained by Voss et al., 2010). Although, a more thorough examination of this theory is needed, it does promise to help us understand the processes underpinning sense of agency in health and disease.

## Why Does Sense of Agency Matter?

The previous section provides an overview of sense of agency research and theory. However, from this overview it would not be entirely clear why any of this matters, particularly from an impact point of view. In the following section I want to address this. I will look at the possible impact of sense of agency research in the context of health and well-being, human-computer-interaction, and the broader issues of free will and responsibility.

### Health and Well-being

#### Schizophrenia and Other Disorders

Schizophrenia is the classic disorder of sense of agency and has been the subject of more agency research than any other disorder. The symptoms of schizophrenia are grouped into two categories: ‘positive symptoms’ and ‘negative symptoms.’ Negative symptoms are defined by the absence of a normal function (for example, ‘alogia’ or reduced speech). Positive symptoms, on the other hand, are defined by the abnormal presence of perceptions (hallucinations) or beliefs (delusions). Abnormal experiences of agency fall within the positive symptom category. Although these abnormal experiences can take many forms, the most common are passivity symptoms (or delusions of control). A patient with passivity symptoms will feel as though his or her actions are not under their control. You can see this in the following patient reported by Mellor (1970, p. 18): ‘It is my hand and arm which move, and my fingers pick up the pen, but I don’t control them. What they do is nothing to do with me.’

Research on patients with schizophrenia has confirmed that these individuals have agency processing problems. In one relatively early study by Daprati et al. (1997), healthy controls and patients with schizophrenia made simple hand movements. They did not directly see their own movements. Instead they saw visual feedback of the movement on screen via a video link. These movements were either (a) their own actual movements, (b) the same movements made by an experimenter in another room, or (c) the movement of that experimenter performing a different movement. The participants and the experimenter were wearing gloves to prevent any visual identity clues. After each trial the participant simply had to say whether the movement on the screen was their own movement or the experimenter’s. Compared with controls, patients – especially those experiencing passivity symptoms – made more errors in attributing the action to its correct source when the experimenter made the same movements as them. In this situation of agentic uncertainty, patients struggled to recognize their own movements.

These action recognition problems have since been confirmed in a number of other studies. For example, Franck et al. (2001) tested patients and healthy controls on an action recognition task. In this experiment they made movements and again only saw video feedback of the movement. In one condition different levels of spatial distortion were introduced. In another condition different time delays were introduced. After each trial participants had to say whether the hand movements on the screen matched their own. Healthy participants tended to say no earlier in both conditions than patients who took much longer to detect these mismatches. Again this suggests abnormal action awareness in patients.

Where agency research on patients has been particularly useful is in uncovering the information processing abnormalities underpinning these disordered experiences of agency. Patients with schizophrenia seem to have specific problems with sensorimotor prediction, which, as we saw in the previous section, is crucial for the sense of agency. One line of evidence comes from studies on sensory attenuation. The neural response to sensory feedback generated by a voluntary action is attenuated – the brain cares less about the things it can predict (Blakemore et al., 1999). This can explain our inability to tickle ourselves: self-tickling is less effective because we can predict the sensory consequences of our actions, resulting in the sensory percept being attenuated. Interestingly, patients with schizophrenia *can* tickle themselves (Blakemore et al., 2000). This finding strongly suggests that patients struggle predicting the sensory consequences of their actions.

Another line of evidence comes from research on intentional binding. As we saw in the previous section, voluntary actions are associated with a compression of the perceived interval between the action and its effect: the perceived time of action is shifted toward the effect, and the perceived time of the effect is shift back toward the action. In a study on healthy adults, Moore and Haggard (2008) showed that the action component of the binding effect (the shift in perceived time of action toward the outcome) is partly linked to prediction. When the outcome was probable, but not always present, there was a shift in perceived time of action even on those trials where the outcome did not occur. This was not the case when the outcome was unpredictable. In follow-up work by Voss et al. (2010), it was found that this predictive effect was absent in patients with schizophrenia.

These problems with prediction can help us explain the behavioral findings from individuals with schizophrenia that I described above. For example, Franck et al. (2001) found that patients struggled to detect temporal and spatial discrepancies between their movements and the feedback of those movements. If the patient struggles to predict *where* their hand should be during movement, they will struggle to detect spatial distortions. Furthermore, if the patient struggles to predict when their hand should move, they will struggle to detect temporal delays.

More recently it has been suggested that problems with prediction represent a core deficit in the disorder (Fletcher and Frith, 2009). On this view predictive deficits can explain positive symptoms more generally, not just passivity symptoms. Clearly we need to find out more about the nature and origins of this predictive deficit in individuals with schizophrenia, but it at least offers us a starting point in the quest to understand and ultimately treat the disorder. Developing interventions to remedy these agency processing problems is one possible avenue for impact.

Aberrant experiences of agency are not just confined to patients with schizophrenia. Indeed, aberrant experiences of agency can be seen in various disorders. Anosognosia for hemiplegia is one such disorder, and is attracting growing interest in the field. ‘Anosognosia’ comes from Greek words nosos (meaning “disease”), and gnosis (meaning “knowledge”), so patients with anosognosia are unaware of their disease or impairment. There are many kinds of anosognias, but the most relevant for us is anosognosia for hemiplegia. These are patients who are paralyzed, usually following stroke, but who are unaware of this impairment. The following description from Berti et al. (2007) is of a patient with anosognosia for hemiplegia:

“CR presented severe and persistent anosognosia for her left hemiplegia... She never spontaneously reported her motor problems. When questioned about her left arm, she always claimed that it could move without any problem. When asked to actually perform movements, she attempted to perform the action, and after a few seconds she appeared to be satisfied with her performance” (p. 172).

From an agency point of view this disorder is intriguing. It suggests that an individual can experience a sense of agency for movements that they cannot make, and for which there is compelling sensory evidence to confirm their paralysis. Research carried out by Fotopoulou et al. (2008) shows that patients do in fact discount sensory evidence in their agency assessments. When instructed to make a movement, they will claim to have moved despite contradictory visual feedback. What this implies is that the experience of agency in these individuals is strongly governed by pre-motor agency cues, such as intentions and sensorimotor predictions. As with the schizophrenia patients, we clearly we need to find out more about the exact nature of this deficit, but it again gives us a useful starting point for the development of therapeutic interventions. For example, it might be useful to try to find ways of increasing the weighting that anosognosia for hemiplegia patients give to sensory feedback, either through cognitive/behavioral interventions or through neural interventions (e.g., pharmacological).

Beyond anosognosia for hemiplegia and schizophrenia there are a number of other disorders that are beginning to attract interest from agency researchers. In Obsessive Compulsive Disorder, for example, it has been shown that patients have deficits in sensorimotor prediction resulting in a reduction of sensory suppression (Gentsch et al., 2012). This finding echoes those from patients with schizophrenia described above. It has also been shown that individuals with high obsessive-compulsive tendencies tend to omit agency from spoken language, perhaps indicating a reduced sense of agency in these individuals (Oren et al., 2016).

From this brief (and far from complete) survey of the clinical research on sense of agency, it should be apparent that aberrant experiences of agency are strikingly common in a range of different disorders. In the field of schizophrenia research, some have claimed that such disturbances in self-awareness are, in fact, a core feature of the disorder (e.g., Sass, 2014). It is a question for future research to find out whether we should also start thinking about certain other disorders in the same way. Whether or not this comes to pass, it is now incumbent on agency researchers to use these findings from patients with disorders of sense of agency to begin developing interventions aimed at remedying them.

#### Healthy Aging

With an aging population there has been a strong push to understand cognitive changes across the lifespan. This is with a view to mitigating some of the negative effects of these changes in older adults. Changes in sense of agency seem to be a feature of old age and therefore warrant further investigation. Understanding these changes, and developing interventions aimed at remedying them could serve to improve well-being in older adulthood.

The majority of work on sense of agency in old age has focussed on examining the link between general changes in sense of agency (based on self-reports) and old age, and how these relate to various indices of health and well-being. Based on this work it is clear that old age is associated with a reduction in the sense of agency. For example, in a large-scale survey of Americans, Lachman and Firth (2004) found that 62% of older adults disagreed with the statement “What happens in my life is beyond my control” whereas almost 80% of young adults (25–39 years) disagreed with it. This reduction begins at around the age of 50 years and continues into older adulthood, with the most rapid decline occurring between 60 and 80 years (Mirowsky, 1995). Importantly, this reduction in sense of agency is associated with poor health and a reduction in quality of life (Langer and Rodin, 1976; Rodin and Langer, 1977), which itself highlights the pressing need for rigorous experimental research.

A key factor in this reduced feeling of control is likely to be a reduction in the basic capacity for agency due physical impairment (Mirowsky, 1995). However, there might also be neurocognitive factors underpinning this reduction in the sense of agency. To-date, few studies have directly examined this from an experimental psychology or cognitive neuroscience perspective. One of the few that have is a study by Metcalfe et al. (2010). They found that the experience of control in older adults less sensitive to three external performance manipulations (such as the insertion of a temporal delay between their movement and a cursor moving on the screen) than a control group of younger adults. The pattern of results is intriguing, suggesting that older adults have a reduced sensitivity to external sensory cues to agency. Future research should explore this in more detail. By uncovering the agency processing abnormalities in older adults it will then be possible to start developing interventions aimed at remedying them.

### Applications Beyond Health and Well-being

The potential impact of agency research extends beyond health and well-being. In this section I consider two of the areas where agency is (or should be) having an impact.

#### Human-Computer-Interaction (HCI)

Most of us spend a lot of time interacting with computers, both for work purposes and for social and leisure purposes. Perhaps because of the ubiquity of our interactions with computers we tend not to spend much time thinking about them (unless things go wrong or we buy a new computer/adopt a new operating system). Another reason why these interactions often go unnoticed might be that a lot of thought goes into designing the interface that sits between the computer and the user. User experience is at the heart of interface design, and this is where sense of agency comes in.

It has long been recognized that the user’s sense of agency is an important consideration when designing new interfaces. Indeed, the seventh of Shneiderman’s Eight Golden Rules of Interface Design states that designers should create interfaces that “support an internal locus of control” (Shneiderman, 1992). This is based on the idea that users “strongly desire the sense that they are in charge of the system and that the system responds to their actions” (Shneiderman, 1992). In light of this, interface design will benefit greatly from scientific research on sense of agency – both in terms of measures that have been developed and in the understanding of what neurocognitive processes shape sense of agency.

Some researchers are already trying to bridge the gap between HCI and sense of agency. In previous work we have looked at how different user interfaces impact on the user’s sense of agency. In one study (Coyle et al., 2012) we compared intentional binding for traditional keyboard input with intentional binding for a novel input modality called ‘skinput’ (whereby a user controls the computer by tapping on their own skin). We found that intentional binding was stronger for skinput, suggesting that this input modality increases the user’s sense of agency. In another study (Limerick et al., 2014) we measured intentional binding for a speech interface. We found that intentional binding was significantly reduced for the speech interface compared with the keyboard interface. Findings like these are potentially useful for those working in interface design. We have shown the utility of intentional binding as a measure of sense of agency – this may offer a more rigorous measure of the user’s sense of agency than currently used measures. We have also shown that different modalities are associated with differences in sense of agency. Given the importance of sense of agency for interface design, this is information is potentially useful – for example, when it comes to speech, our findings may help explain the difficulty and unpopularity of speech interfaces (Aylett et al., 2014).

Interfaces such as skinput and speech require the user to make an over behavioral response. In brain-machine-interfaces (BMIs), there is no such requirement. As such, BMIs are a particularly exciting development in terms of our interaction with computers. By exploiting relatively recent advances in the acquisition and analysis of neuroimaging data, these technologies allow users to interact with computers and other devices without the need for overt behavioral responses. Of particular interest here is the user’s experience of agency given the absence of overt behavior. This was investigated by Evans et al. (2015). They found that agency judgments were modulated in a way that was similar to what one would expect for bodily movements, suggesting that BMIs can generate experiences of agency in users. Despite these similarities, they also observed that the effect of manipulated visual feedback in these tasks had a more pronounced effect on the user’s sense of agency than one would expect with bodily movements. This finding is consistent with the cue integration theory (e.g., Moore and Fletcher, 2012) as it shows that in the absence of strong internal motor cues, external visual feedback dominates.

A more pressing practical concern in the context of HCI is the user’s sense of agency when interacting with automated technologies. Indeed, an increasing number of our interactions with computers and technology are being automated, with the system taking over a lot of the control that would have been in the hands of the user. Examples of automation include things like auto-correct in word processors through to driverless cars. Automation raises a number of issues when it comes to sense of agency; the most of obvious is the potential loss of sense of agency in the user. This is important if we remind ourselves of the prominence that the user’s sense of agency has in the design of interfaces.

One area where automation is well-established is aircraft control – much of the pilot’s work is now carried out by a computer. Berberian et al. (2012) examined the sense of agency under different levels of automation in a flight simulator. They found that increasing automation reduced sense of agency. This finding is important, especially if sense of agency is linked to performance (something which is not yet established but would be interesting to look at). It is also important in situations where the automated system goes wrong – the attribution of responsibility in these situations, which is incredibly important socially and legally, might be guided by findings such as these.

#### Freedom and Responsibility

The attribution of responsibility, something that I touched on at the end of the previous section, is one of the key social functions of sense of agency (Frith, 2014). Humans seem to place a premium on responsibility – most, if not all, societies require that their members are held responsible for what they do. Haggard and Tsakiris (2009) have argued persuasively that sense of agency plays a key role in guiding attributions of responsibility. For Frith (2014) this bearing of responsibility for one’s own actions plays an important social function. It means that people can be held account for what they do which in turn allows behavior to be legitimately managed through punishment or reward. This behavioral management can be implemented so as to benefit the social group and promote social cohesion. In many societies the legal system is a tool which facilitates this kind of behavioral management. Given the importance of sense of agency for establishing responsibility, research in this area is therefore likely to have implications for the legal system.

Responsibility is closely related the concept of free will. That is, for most people it only makes sense to hold someone responsible for their actions if they are freely in control of them. Indeed, the *The Oxford English Dictionary* defines free will as ’the power of an individual to make free choices, not determined by divine predestination, the laws of physical causality, fate, etc. Also: the doctrine that human beings possess this power and are *hence able to direct and bear responsibility for their own actions’* (emphasis added).

Free will is the elephant in the room when it comes to sense of agency research. Researchers tend to side step the issue of free will and instead focus solely on uncovering things like the neurocognitive basis of agentic experience. That is, whether or not we have free will, we unquestionably *do* have the experience of agency when we make actions and scientific research has tended to focus on understanding this experience. This evasion of the free will debate is understandable; philosophical debates on free will are often quite complex and confusing, especially for scientists with no background in philosophy. However, I think those of us working on this topic should try to engage more with this debate. In terms of impact, the social and legal consequences of this debate are immense, and our findings should be helping to inform this debate.

Nichols (2011) has highlighted an interesting point of contact between sense of agency research and the free will debate. The free will problem arises because on the one hand we feel like conscious, rational free agents, and yet we recognize that this is incompatible with determinism. The relevance of sense of agency to this issue is that it is these experiences of agency surrounding our voluntary actions that give rise to the general feeling that we are conscious, rational free agents. According to Nichols, understanding the neurocognitive origins of free will beliefs will not tell us if they are true or not, but will help us evaluate whether or not those beliefs are justified. Although, this is just one of many possible links between free will and sense of agency, it does offer a potentially useful starting point for bringing the two fields together.

There are of course some scientists who are willing to engage with the free will debate, and they are to be commended (see, for example, Wegner, 2002; Gazzaniga, 2011). However, these contributions can often take the form of what many philosophers regard as overly strong claims about our lack of free will. Mele (2014), for example, argues that many scientists who make such claims have a conception of free will that most philosophers, and indeed a lot of the general public, would not subscribe to (see also Dennett, 1984, 2004, for extensive discussions of different conceptions of free will). Other philosophers have contested this scientific work on different grounds. For example, Gallagher (2008) suggests that the scientific challenges to free will are misguided because they confuse the issue of mental causation (free will), with the issue of motor control. Because of this, these experiments can tell us something about motor control, but not free will. These examples from the philosophical literature show that if we scientists who work on sense of agency are to contribute meaningfully to the free will debate, there is a need for us to be more engaged with the philosophical work on the topic.

## Concluding Remarks

In line with the stated aims of this article, I hope that I have provided the reader with a useful overview of the literature on sense of agency. I also hope that I have given the reader a sense of the potential importance and impact of this research. Much of the applied work is still in its infancy and I have only scratched the surface in terms of the potential applications of agency research. It is now up to us to drive this forward and to make a concerted effort to translate the findings of our more basic research on sense of agency into useful and effective applications.

## Author Contribution

The author confirms being the sole contributor of this work and approved it for publication.

## Conflict of Interest Statement

The author declares that the research was conducted in the absence of any commercial or financial relationships that could be construed as a potential conflict of interest.
